# ASO Author Reflections: Real-World Conclusions on Locoregionally Recurrent Colon Cancer

**DOI:** 10.1245/s10434-022-12772-x

**Published:** 2022-11-05

**Authors:** Hidde Swartjes, Hans de Wilt

**Affiliations:** Department of Surgery, Radboud Institute for Health Sciences, Radboud University Medical Center, Nijmegen, The Netherlands

## Past

With a reported median overall survival of 9 months, locoregionally recurrent colon cancer (LRCC) is an undesired outcome after curative treatment of primary non-metastasized colon cancer.^1^ The disease, defined as recurrent colon cancer near the site of the primary resection or in regional lymph nodes, is diagnosed in approximately one in ten patients during the 5 years after curative treatment.^1,2^ Disease presentation is heterogenous between patients, and consensus on the optimal treatment is nonexistent. Evidence-based decision-making is complicated by the limited literature on the disease: the scarce amount of literature primarily consists of studies with small numbers of included patients, studies conducted in single centers, or studies from over a decade ago.

## Present

Our population-based, retrospective cohort study provides a state-of-the-art overview of LRCC in the Netherlands.^3^ More than 3500 patients with primary non-metastasized colon cancer were included in this nationwide study. With a 3-year cumulative incidence of 3.8%, the incidence of LRCC was relatively low. Approximately two-thirds (62.7%) of the patients with LRCC presented with synchronous distant metastases. Patients with primary colon cancer and an age of 70 years and older, incomplete resection margins, pT4 tumors, and pN1–2 lymph node metastases were at an increased risk for development of LRCC. On the contrary, adjuvant chemotherapy seemed protective for the development of LRCC in pT4N0 or pTXN1-2 patients with colon cancer [hazard ratio (HR) 0.47, 95% confidence interval (CI) 0.31; 0.93). Curative-intent treatment was given to 22.4% of patients with LRCC, mainly comprising patients without synchronous distant metastases. Three-year overall survival in patients who received curative-intent treatment was high (71%).

## Future

The comprehensive results from our study clearly contribute to the literature on the epidemiology, management, and outcomes of LRCC. Population-based overall survival outcomes after curative-intent treatment of LRCC were higher than expected. We hypothesize that adequate patient selection for curative-intent treatment through multidisciplinary team meetings—a common practice in the majority of Dutch hospitals—has contributed significantly to this high overall survival statistic. Nonetheless, questions on which patients with LRCC should receive which type of treatment (e.g., “Should we perform surgery?”, “Should we perform local treatment of distant metastases?”, “Should we give additional chemotherapy?”) could not be answered by our study. Future research, preferably in the form of international collaborations aimed to include high numbers of patients with LRCC, will further decrease the knowledge gap on LRCC.
